# Trait-anger, hostility, and the risk of incident type 2 diabetes and diabetes-related complications: a systematic review of longitudinal studies

**DOI:** 10.1192/j.eurpsy.2022.1144

**Published:** 2022-09-01

**Authors:** M. Mohseni, N. Lindekilde, G. Forget, R. Burns, N. Schmitz, F. Pouwer, S. Deschenes

**Affiliations:** 1 University College Dublin, Psychology, Belfield, Ireland; 2 University of Southern Denmark, Psychology, Odense, Denmark; 3 Carleton University, Psychology, Ottawa, Canada; 4 University of Tübingen, Medicine, Tubingen, Germany

**Keywords:** hostility, diabetes, systematic review, Anger

## Abstract

**Introduction:**

There is a well-established association between anger, hostility, and an increased risk of cardiovascular disease. Emerging evidence also suggests associations between anger/hostility and type 2 diabetes (T2D), though evidence from longitudinal studies has not yet been synthesized.

**Objectives:**

To systematically review findings from existing prospective cohort studies on trait anger/hostility and the risk of T2D and diabetes-related complications.

**Methods:**

Electronic searches of MEDLINE (PubMed), PsychINFO, Web of Science, and CINAHL were performed for articles/abstracts published up to December 15, 2020. Peer-reviewed longitudinal studies conducted with adult samples, with effect estimates reported for trait anger or hostility and incident T2D or diabetes-related complications, were eligible for inclusion. Risk of bias/study quality was assessed. The review protocol was published a priori in PROSPERO (CRD42020216356) and was in keeping with PRISMA guidelines. Screening for eligibility, data extraction, and quality assessment was conducted by two independent reviewers.

**Results:**

Four studies with a total of 155,146 participants met the inclusion criteria. A narrative synthesis of extracted data was conducted according to the Synthesis Without Meta-Analysis guidelines. While results were mixed, our synthesis suggested a positive association between high trait-anger/hostility and increased risk of incident T2D. No longitudinal studies were identified relating to anger/hostility and incident diabetes-related complications. Geographical locations of the study samples were limited to the USA and Japan.

**Conclusions:**

Further research is needed to investigate whether trait-anger/hostility predicts incident type 2 diabetes after adjustments for potential confounding factors. Longitudinal studies are needed to investigate trait-anger/hostility and the risk of diabetes-related vascular complications.

**Disclosure:**

No significant relationships.

